# Giant ultrafast dichroism and birefringence with active nonlocal metasurfaces

**DOI:** 10.1038/s41377-024-01545-8

**Published:** 2024-08-23

**Authors:** Giulia Crotti, Mert Akturk, Andrea Schirato, Vincent Vinel, Anton A. Trifonov, Ivan C. Buchvarov, Dragomir N. Neshev, Remo Proietti Zaccaria, Paolo Laporta, Aristide Lemaître, Giuseppe Leo, Giulio Cerullo, Margherita Maiuri, Giuseppe Della Valle

**Affiliations:** 1 Department of Physics, Politecnico di Milano, 20133 Milano, Italy; 2 Istituto Italiano di Tecnologia, 16163 Genova, Italy; 3 Laboratoire Matériaux et Phénomènes Quantiques (MPQ), Université Paris Cité & CNRS, 75013 Paris, France; 4 John Atanasoff Center for Bio and Nano Photonics (JAC BNP), 1164 Sofia, Bulgaria; 5 Department of Physics, St. Kliment Ohridski University of Sofia, 5 James Bourchier Boulevard, 1164 Sofia, Bulgaria; 6 ARC Centre of Excellence for Transformative Meta-Optical Systems (TMOS), Research School of Physics, Australian National University, Acton, ACT 2601 Australia; 7 Cixi Institute of Biomedical Engineering, Ningbo Institute of Industrial Technology, Chinese Academy of Sciences, Ningbo, 315201 China; 8 Istituto di Fotonica e Nanotecnologie (IFN), Consiglio Nazionale delle Ricerche, 20133 Milano, Italy; 9 Université Paris-Saclay, CNRS, Centre de Nanosciences et de Nanotechnologies, 10 Boulevard Thomas Gobert, 91120 Palaiseau, France; 10 Institut Universitaire de France (IUF), Paris, France

**Keywords:** Metamaterials, Photonic devices, Optical spectroscopy

## Abstract

Switching of light polarization on the sub-picosecond timescale is a crucial functionality for applications in a variety of contexts, including telecommunications, biology and chemistry. The ability to control polarization at ultrafast speed would pave the way for the development of unprecedented free-space optical links and of novel techniques for probing dynamical processes in complex systems, as chiral molecules. Such high switching speeds can only be reached with an all-optical paradigm, i.e., engineering active platforms capable of controlling light polarization via ultrashort laser pulses. Here we demonstrate giant modulation of dichroism and birefringence in an all-dielectric metasurface, achieved at low fluences of the optical control beam. This performance, which leverages the many degrees of freedom offered by all-dielectric active metasurfaces, is obtained by combining a high-quality factor nonlocal resonance with the giant third-order optical nonlinearity dictated by photogenerated hot carriers at the semiconductor band edge.

## Abstract (web summary)

“Upon ultrafast photoexcitation, an all-dielectric nonlocal metasurface enables giant dichroism and birefringence modulations, working as a quarter-waveplate actively reconfigurable over picoseconds.”

## Graphical abstract

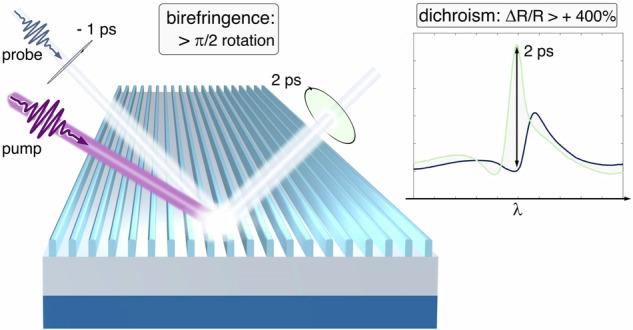

## Introduction

A plethora of light-matter interaction phenomena occurring across physics, chemistry or biology are intrinsically polarization dependent. As such, light polarization is a pivotal degree of freedom to exploit both for applications and fundamental investigations. Active (i.e. transient and reversible) and ultrafast polarization control would allow high-speed data encoding and signal processing (both from a classical^1^ and quantum information^2^ perspective), e.g. for free-space optical links transmitting optical bits (even in the visible/near-infrared) at GHz rates, as well as the modulation of pseudospin properties for the development of advanced quantum electronic devices^3^. Similarly, it could be possible to implement THz speed tuning or switching of material processes such as lattice excitations^4^ or modifications of molecular dynamics^5,6^. Additionally, the study of enantiomers in organic chemistry, and in general the control and detection of chiral systems and chiroptical effects^7^, which are ubiquitous in biology^8^ and can be also inorganic^9^ or hybrid^10,11^, would benefit from the capability of manipulating polarization on sub-picosecond timescales^12^.

To overcome the fundamental speed limits of electro-optical approaches, an all-optical paradigm for polarization control has been proposed. It consists in triggering a third-order nonlinearity in an active medium by an ultrashort control laser pulse, which transiently modulates the material permittivity experienced by a low-intensity (probe) beam. By carefully engineering the material platform, it is thus possible to achieve control of light by light. Many approaches have been proposed for the all-optical manipulation of light’s fundamental degrees of freedom^13–20^. Active tailoring of amplitude, phase and polarization has been demonstrated in a variety of systems, from semi-metal thin films^21^, to nonlinear metamaterials based on plasmonics^22–24^ and epsilon-near-zero architectures^25^.

Despite these recent exciting advancements, practical applications of all-optical polarization control require extreme flexibility of the selected structure, a higher compactness (with close to normal-incidence operation for better alignment) and superior modulation efficiency (of the order of 100% under a control beam fluence one or two orders of magnitude lower than the typical damage threshold of ~mJ cm^-2^). A possible route towards the ultimate limit of photonic integration has been disclosed by flat optics^26^, specifically by resorting to photonic metasurfaces^27–36^. These are quasi two-dimensional arrangements of resonant nanostructures, packed in subwavelength configurations. By exploiting optical resonances — which concentrate fields locally, at the individual scatterer scale – it is possible to achieve high levels of field enhancement, thus strongly outperforming unpatterned thin films. Moreover, flexibility is granted by the large number of geometrical degrees of freedom, to be leveraged during the design process.

In the last decade, high-index semiconductor-based architectures have attracted increasing interest for all-optical modulation^37–39^. Indeed, they present several advantages^40^: on one hand, they enable the onset of high quality factor resonances, including magnetic Mie-type resonances^41–43^ and quasi-bound states in the continuum (BICs)^44,45^. Furthermore, semiconductors feature lower absorption losses compared to plasmonic materials. Most importantly, one of the physical phenomena presiding over the optical nonlinearity of semiconductors is the photogeneration of free carriers, which is very efficient for direct bandgap materials^38^. Throughout their relaxation, these photoexcited carriers drive a transient permittivity modulation via different effects^46^, encompassing Drude-like plasma formation, band filling and bandgap renormalization. By tuning several parameters (e.g., the operating spectral range or the pump fluence), the interplay of these mechanisms and their relative weights can be regulated.

We have investigated these aspects in recent studies^47,48^ on Aluminum Gallium Arsenide (AlGaAs) nanoantennas for nonlinear applications. The key role of the band filling effect was highlighted when operating at energies near the bandgap of the material, with very efficient modulations of the permittivity. Interestingly, band filling is responsible for a purely real modification of permittivity at energies below the bandgap, whereas it also entails a *negative* imaginary part contribution for probe energies above bandgap (that is, describing a reduction of loss channels, possibly a transient gain)^46^. The effect of such a mechanism on a macroscopic optical observable (for instance reflection or transmission) has not been explored, since research on similar dielectric nanostructures has mainly focused on the transparency window of the semiconductors^38^.

Here, we theoretically predict and experimentally demonstrate, using ultrafast pump-probe spectroscopy, giant all-optical dichroism and birefringence modulation in a custom designed AlGaAs-based metasurface. By tuning free-carrier permittivity modulation at the band edge, and a polarization-selective nonlocal resonance at bandgap, we obtain unprecedented polarization modulation efficiency at moderate excitation levels. Specifically, we observe up to 470% differential reflection in the dichroism experiment, with control beam fluence of 70 μJ cm^-2^; up to a π/2 transient phase shift between the components, in the birefringence experiment, with control fluence of 180 μJ cm^-2^. Numerical simulations, based on a multi-step procedure including dynamic modelling of free-carriers and full-wave electromagnetic computations for the optical response, are in good agreement with experimental data. This allows us to disentangle the physical phenomena taking place after photoexcitation, pinpointing the synergy of band filling with the resonant response of the structure as responsible for the excellent modulation performance.

## Results

**Fig. 1:**
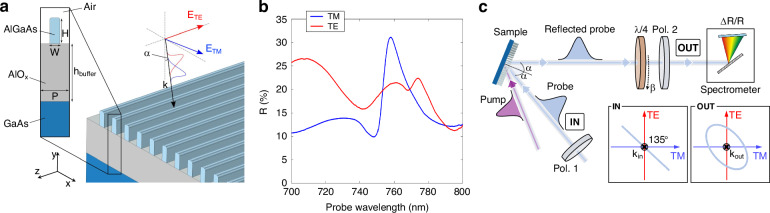
Metasurface characterization and experimental setup. **a** Sketch of the sample and the probe illumination conditions for both static reflection measurements and dichroism experiment. Left inset represents the *x-y* plane cross-section of the unit cell of the grating, with its relevant geometric parameters: wire width and height (nominal values *W* = 165 nm and *H* = 400 nm, respectively), periodicity (*P* = 450 nm), height of the AlO_x_ buffer (*h*_buffer_ = 900 nm). The scheme on the right shows the direction of the incident beam wave-vector **k**_in_, and the unit vectors representing the TE and TM directions (red and blue, respectively). The angle of incidence *α* is ~9°. **b** Static reflectance of the metasurface upon illumination with either TE- (red) or TM-polarized (blue) light. **c** Sketch of the polarization-resolved pump-probe apparatus. The pump pulse is tuned at 400 nm and a broadband probe pulse spans the 700– 800 nm spectral region. In the dichroism experiment, a first polarizer (Pol.1) is set either along TM or TE direction, namely at 0° or 90° in the polarization plane (defined as IN plane orthogonal to the **k**_in_ propagation direction, inset); a second polarizer (Pol.2) is placed on the reflected probe path following the same directions 0° or 90° in the polarization plane (defined as OUT plane orthogonal to the **k**_out_ propagation direction, inset). For the birefringence experiment, a quarter-waveplate (λ/4) is placed before Pol.2. In this case, Pol. 1 and Pol. 2 are set at 135°, while the quarter-waveplate fast axis is rotated at an angle β, different for each of the four measurements needed for polarization reconstruction

We designed a metasurface consisting in an array of Al_.18_Ga_.82_As nanowires on top of a ~ 900 nm AlO_x_ layer with a GaAs substrate, as depicted in Fig. 1a. The fabrication procedure is detailed in the Supporting Information section S1. The inset on the left shows the unit cell cross-section in the *x–y* plane, with the relevant geometrical parameters—wire width *W* and height *H*, periodicity *P*.

On the right of Fig. 1a, the probe illumination conditions for the static characterization and dichroism experiment are sketched: the beam is impinging on the sample with an angle of incidence *α* ~ 9°, and it is polarized either in the *x*–*y* plane (i.e., perpendicular to the wires, TM polarization) or along the *z*-axis (parallel to the wires, TE polarization). The measured reflectance spectra in unperturbed (static) conditions for TE and TM polarizations are reported in Fig. 1b. The anisotropy of the sample affects the optical response, granting a strongly dichroic behavior. For TM polarization, a sharp resonance is found at 758 nm, just below the bandgap, which is at 750 nm for these values of Al concentrations^49^. This is an extended-state resonance (see Supporting Information section [S2]), enabled by the metasurface configuration designed to provide a nonlocal response. Instead, the TE reflectance presents a narrow asymmetric peak, which can be identified as a quasi-BIC state, at slightly longer wavelengths (~770 nm). These features match our needs towards an efficient switching and synthesis of light polarization, since, according to the metasurface design, they are located precisely in the spectral region of interest, near the AlGaAs bandgap.

We used polarization-resolved ultrafast pump-probe spectroscopy to characterize transient dichroism and birefringence. In the first experiment, we measured the differential reflection, Δ*R*/*R* = (*R’*-*R*)/*R*, where *R’* and *R* are the reflectance spectra of the perturbed (after pump) and unperturbed (pump is not applied) metasurface, for both TM and TE probe polarizations. A comparison between Δ*R*/*R* maps allows to assess the efficiency of the platform in modulating its dichroic properties.

To illustrate the capability of birefringence modulation, in a second experiment we set a mixed polarization for the incident probe beam (precisely, it is linearly polarized at 135° in the plane defined by the TM and TE directions, see below for details). Then, a modified detection line, including a quarter-waveplate and a polarizer, enables us to perform an ultrafast polarimetry measurement, aimed at investigating the transient modulations of the polarization ellipse of the reflected light.

A simplified scheme of the experimental setup is presented in Fig. 1c. Notice that the sketch describes the optical components used for the birefringence experiment, while for the dichroic experiment the quarter waveplate (λ/4) is removed. Details of the apparatus can be found in the Methods section.

### Dichroism

**Fig. 2:**
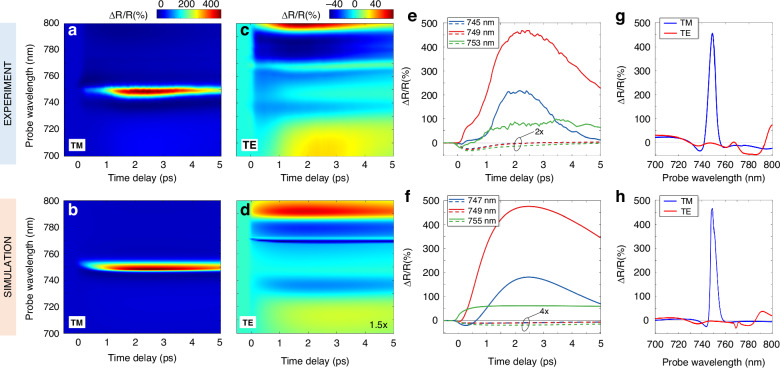
Modulation of dichroism. **a**, **b** Experimental (**a**) and simulated (**b**) TM differential reflectance as a function of time delay and probe wavelength. The maps share the same colorbar (top). The pump pulse is centered at 400 nm, it has a duration of 100 fs FWHM, and its fluence is 70 μJ cm^-2^. **c**, **d** Same as (**a**, **b**), respectively, for the TE probe polarization. Data are rescaled in the simulation (**d**) for better reading. **e**, **f** Temporal dynamics of the experimental (**e**) and simulated (**f**) maps at different probe wavelengths. Solid (dashed) lines correspond to TM (TE) signals. **g**, **h** Experimental (**g**) and simulated (**h**) *ΔR/R* spectra at 2 ps pump-probe delay

Results of the ultrafast dichroism experiment are summarized in the upper panels of Fig. 2. Δ*R*/*R* maps for TM and TE polarized probe (panels 2a, 2c) demonstrate that optical pumping produces a remarkable enhancement of the dichroic response of the sample. The spectra reveal an anisotropic modulation: on one hand, we observe a giant signal (up to 470%) in the TM case in a narrow band around 750 nm. This is a genuine property of the transient optical response, as it corresponds to a giant photoinduced increase of the reflectance from the static value, *R* ~ 10% (refer to Fig. 1b) to a transient value of almost 60%. On the other hand, TE reflectivity is also modulated, but only up to a maximum Δ*R*/*R* of ~70%, with complex and varied broadband features, and a relatively low value of about -10% at 748 nm, where the TM reflectivity modulation is peaked. This indicates a giant ultrafast transient dichroism at ≈ 750 nm wavelength under a pump fluence *F* = 70 μJ cm^-2^, which is low for semiconductors^38,47,48^. The reflectivity modulation peak of ~470% is up to 5 times higher than in previous studies^38^ and is achieved at a much lower pump fluence; more importantly, the fluence here employed is at least one order of magnitude below the damage threshold, which for AlGaAs is ~ 1 mJ cm^-2^.

These remarkable features are even more evident when examining temporal and spectral sections of the maps of Fig. 2. Figure 2e shows the temporal dynamics of Δ*R/R* at selected wavelengths for the TM (solid lines) and TE (dashed lines) polarization. The modulation peak is not instantaneous but is reached at 2 ps delay with respect to the arrival of the pump pulse. A Δ*R*/*R* spectrum at this time delay is presented in Fig. 2g. As expected, there is a close relation between the spectral positions of the unperturbed resonances shown in Fig. 1b and the peaks (dips) in the Δ*R*/*R* spectrum.

To interpret these results, we applied a multistep semiclassical modeling approach^48^. Here we briefly describe the physical processes contributing to the optical nonlinearity (see the Methods section and the Supporting Information section S4 for further details). The 400-nm pump pulse excites AlGaAs above its bandgap, producing free carriers. Since the nanowires are remarkably thick (*H* = 400 nm) compared to the skin depth of AlGaAs at the pump photon energy (~16 nm)^48^, the absorption is spatially inhomogeneous. Electron-hole pairs generation is concentrated in hot-spots at the top of the structure, and diffusion processes take place within the first few picoseconds after pump arrival^48^. Then, nonradiative relaxation of carriers occurs, mainly through a trap-assisted recombination process mediated by surface defect states. This mechanism dominates over the Auger recombination (which is the most relevant in bulk configurations), thanks to the wire high surface-to-volume ratio^38^. Conservation of energy implies that recombination events are associated with the emission of phonons, increasing the lattice temperature.

In these terms, photo-absorption can be described through the evolution in time of three variables: *n*_1_(*t*), carrier density in the hot-spot region, driven by pump-intensity; *n*_2_(*t*), carrier density in the bulk of the wire, increasing gradually in time with diffusion from the skin, and depleting with recombination; *Θ*_L_(*t*), the lattice temperature. A simple rate equation system (three-temperature model, 3TM) describes these dynamics (see Methods section). In turn, each of these internal degrees of freedom is responsible for a transient permittivity change. Free carriers modify both intraband and interband transitions at the sub-picosecond timescale, via a Drude-like and a band filling effect, respectively. The latter can be described as a saturation of absorption channels due to Pauli exclusion principle^46^. A weak probe impinging on the medium cannot promote electrons to the conduction band, since its lower part is already filled following pump arrival. Thus, band filling gives rise to a negative modulation of the imaginary permittivity at energies above the bandgap, as well as a broadband real permittivity modulation highly dispersed across the bandgap. Due to the Kramers-Kronig relations, band filling also modifies the real part of Δε in the band edge wavelength range. Lastly, an increase of the lattice temperature triggers a thermo-optical permittivity variation (to a comparatively negligible extent, given the moderate lattice temperature increase, see section S4.4 in the SI document). Once the permittivity is computed via semiclassical formulas^46–48^, the optical response can be retrieved with full-wave electromagnetic simulations, as a function of both probe wavelength and time.

The results of this model are summarized in the bottom panels of Fig. 2, to be compared with the corresponding measurements in the upper panel. The agreement is excellent for both polarizations (panels 2b-2d), apart from a small underestimation of the Δ*R*/*R* in the TE case. In fact, simulations can quantitatively reproduce both the temporal dynamics—including the delayed peak (Fig. 2f), crucially linked to the free-carriers diffusion time^48^—and the spectral features (Fig. 2h).

**Fig. 3:**
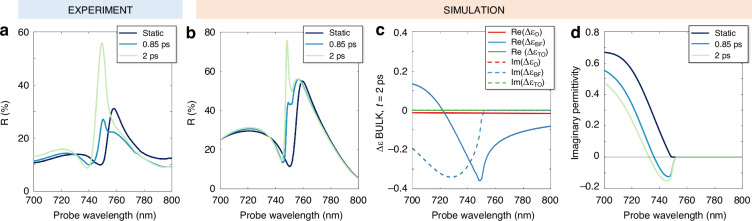
Disentangling contributions to the all-optical modulation. **a** TM reflection in static conditions, at 0.85 ps and at 2 ps (from darker to lighter shades) after pump arrival as directly measured or derived from the experimental results of Fig. 2 (see main text). **b** Simulated TM reflection as in (**a**). **c** Disentanglement of contributions to permittivity variation in the wire bulk at time delay of 2 ps. Red, blue and green lines correspond to Drude (D), band filling (BF) and thermo-optic (TO) effects, respectively. Solid (dashed) lines are relative to the real (imaginary) part modulation. **d** Imaginary part of total permittivity for unperturbed sample and after pump arrival

We also use the model to elucidate the origin of the giant Δ*R*/*R* signal for TM polarization. In Fig. 3a, lines from darker to lighter shade correspond to the TM reflectivity spectrum, respectively, in unperturbed conditions (*R*_static_ as directly measured, see the blue curve in Fig. 1b), at 850 fs and at 2 ps after pump arrival (derived from pump-probe experiments of Fig. 2, using the simple formula *R*(t) = {[Δ*R*/*R*(t)]_measured_ + 1} *R*_static_). The static resonance experiences an ultrafast blueshift, manifested already at 850 fs, in agreement with the evidence that the peak in the Δ*R*/*R* is attained at a wavelength which is ~10 nm shorter than the peak wavelength in the static reflectivity (see Fig. 1b); besides, a reshaping of the peak occurs, with a clear-cut increase of the reflectivity at 2 ps. This underpins the 470% differential signal peak shown of Fig. 2a. Our simulation, in Fig. 3b, predicts the same peculiar evolution in time. To disentangle the effects arising from free carriers and lattice temperature on the optical nonlinearity, we plot the contributions to permittivity variation Δε at the peak of the pump-probe signal, namely at 2 ps, for the interesting spectral range. We report the results in Fig. 3c: solid (dashed) lines correspond to real (imaginary) modulation, whereas color coding marks the Drude, band filling, and thermo-optic contributions. Lattice effects are essentially zero, as expected, since they become significant on the **~**10 ps timescale^48^. Importantly, the permittivity change due to the Drude mechanism is much smaller than the one caused by band filling (up to one order of magnitude at bandgap, for the real part, and almost two orders of magnitude in the high-energy band edge for the imaginary part). Thus, simulations confirm that band filling presides over the most relevant features of the transient optical response in this spectral region, given its vicinity to the material band-edge.

Notice that the real part of the total Δε has a broadband negative sign. This directly translates into a spectral blueshift of the optical response, as observed in the experiment. On the other hand, the imaginary part of Δε has a negative sign for wavelengths up to 750 nm, consistent with a reduction of loss channels. Since the increase of the resonant peak is at a probe wavelength (748 nm) slightly below the bandgap – where the absorption drops – it is crucial to inspect the imaginary part of the total perturbed permittivity, ε(t) = ε^0^ + Δε(t), where ε^0^ is its static value. Figuer 3d shows the imaginary part of the permittivity: Im(ε^0^) along with Im(ε), the latter evaluated at 850 fs and 2 ps. A narrow spectral window (738 – 750 nm) where the sign of ε is negative opens within the first ps, as seen from the dark light blue curve, and broadens for increasing delay (lighter trace): this is a clear-cut mark of the onset of optical gain in this region. In other terms, the photo-injection of carriers through photoexcitation generates a population inversion. Upon arrival of the probe, stimulated emission occurs. The efficient increase in reflection is thus explained as the combination of this phenomenon and the presence of the high quality-factor TM resonance in this precise spectral range.

Considering the complex interplay of mechanisms included in our modeling, the discrepancies between the experimental results of Fig. 3a and the simulations of Fig. 3b are minimal, and of quantitative character only. In particular, the simulations retrieve narrower spectral features compared to the experiment. Also, contrary to the reflectivity peak, whose evolution from 760 nm – 750 nm is well reproduced in the simulations, the simulated spectral dip is always red shifted compared to the experimental one. We attribute such discrepancies to the fact that spatial inhomogeneities, defects and finite size effects are not taken into account in the electromagnetic calculations.

### Birefringence

Results of the birefringence experiment also show prominent features in the 740 nm–750 nm region, caused by the same physical mechanisms, although having a more complex origin and temporal evolution. Indeed, change in the polarization state is an interplay between the modulation of both the amplitude and phase of the two orthogonal components in the polarization plane. To formalize the problem, we employ Jones vector analysis, and describe the incident and reflected waves as follows. For the incident wave, we use a frame of reference analogous to the one schematically sketched in Fig. 1a: the polarization plane, perpendicular by definition to the k-vector (**k**_in_ in Fig. 1c), is spanned by the TM and TE unit vectors ${\hat{{\boldsymbol{u}}}}_{{\bf{TM}}}$, ${\hat{{\boldsymbol{u}}}}_{{\bf{TE}}}$. The probe beam is linearly polarized in the direction of the bisector of the II–IV quadrant of this plane, namely at 135° with respect to the TM direction. Thus, we can write the normalized incident electric field as the Jones vector

$$\left({{E}_{{TM}}^{{inc}}}\atop{{E}_{{TE}}^{{inc}}}\right)=\frac{1}{\sqrt{2}}\left(\begin{array}{c}1\\ -1\end{array}\right)$$

We proceed similarly to define the polarization for the beam reflected by the sample, referred to the plane orthogonal to the appropriate wave vector (**k**_out_ in Fig. 1c), with signs consistent with the right-hand rule for the triplet (**k,**${\hat{{\boldsymbol{u}}}}_{{\bf{TE}}}{\boldsymbol{,}}{\hat{{\boldsymbol{u}}}}_{{\bf{TM}}}$) as before. We can write the normalized reflected field as

$$\left({{E}_{{TM}}^{r}}\atop{{E}_{{TE}}^{r}}\right)=\left({{\rho }_{{TM}}{e}^{i{\varphi }_{{TM}}}}\atop{{\rho }_{{TE}}{e}^{i{\varphi}_{{TE}}}}\right)$$

where ${\rho }_{{TM}}$, ${\rho }_{{TE}}$ are positive real numbers representing the amplitudes, whereas ${\varphi }_{{TM}}$, ${\varphi }_{{TE}}$ are the phases in the (-π,π) interval. Since we aim to demonstrate phase-sensitive functionality such as that of a transient optical waveplate, it is useful to define the relative phase between the components, $\varphi {=\varphi }_{{TM}}-{\varphi }_{{TE}}$, as a figure of merit for the ellipticity of the wave. The pump-probe scheme is the same as in the dichroism experiment, with higher, but still moderate level of excitation (pump fluence of ~180 μJ cm^-2^).

**Fig. 4:**
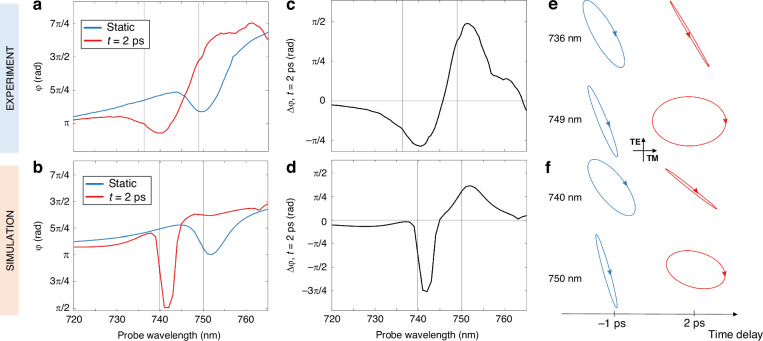
Modulation of birefringence. **a**, **b** $\varphi$ (static conditions, blue curve) and $\varphi {\prime}$ (at 2 ps after pump arrival, red curve), experimental (**a**) and simulated (**b**). **c**, **d** Experimental (**c**) and simulated (**d**) transient phase change $\triangle \varphi =\varphi {\prime} -\varphi$. **e**, **f** Polarization ellipses of the reflected wave at selected wavelengths, as retrieved from measurements (**e**) and simulations (**f**), in static (-1 ps) and perturbed conditions (2 ps). The polarization plane has the TM (TE) direction along the horizontal (vertical) axis

Results of the experiments in terms of $\varphi$ as a function of probe wavelength are reported in Fig. 4, top panels. Specifically, Fig. 4a compares the static ($\varphi$, blue line) and nonequilibrium ($\varphi$’, red line) responses at 2 ps, whereas Fig. 4c shows the variation $\Delta \varphi =\varphi ^{\prime} -\varphi$. A value of $\Delta \varphi$ ~ π/2 implies that, after pumping, the metasurface introduces an additional π/2 phase shift between the TM and TE components. That is, if in static conditions the reflected beam is linearly polarized, in the perturbed case the detected polarization is circular. This is very similar to what happens at 749 nm, as can be seen in Fig. 4e, by inspecting the complete polarization ellipse as retrieved from the measurements. Indeed, pump absorption grants switching of a quarter-waveplate functionality at the red wing of the TM resonance. Instead, at 736 nm, corresponding to the blue-wing of a dip in the $\Delta \varphi$ spectrum, the polarization is efficiently converted from elliptical to almost linear. Simulations (Figs. 4b, 4d and 4f) show a good agreement with experimental results, although predicting a sharper drop in phase at 742 nm, which marks the position of the shifted dip in the TM polarization. Again, this slight discrepancy with experiments is attributed to defects and imperfections in the physical sample, causing broadening and smoothing of the sharpest optical features.

## Discussion

We have presented an all-dielectric metasurface working in reflection and capable of extremely efficient modulation of both dichroism and birefringence. As revealed by our model, these performances are enabled by the tuning of extended state resonances near the bandgap of the semiconductor, which allows full exploitation of the band filling effect due to photogenerated free carriers.

A narrow gain window at the blue edge of the bandgap, opened following pump absorption, also contributes to the achievement of the giant dichroism reported in the experiments. While stimulated emission from optical pumping of semiconductors is well known and has been exploited e.g. in lasing applications^50,51^, also with AlGaAs-based structures^52^, its engineering in an all-optical modulation scheme was still unexplored.

In parallel, the sample birefringence modulation also arises from the outlined physical mechanisms, albeit in a subtler way. Transient phase change between the two polarization components can be ascribed to the free carrier induced blueshift of the optical response (Figs. 4a and 4b). Indeed, the efficient birefringence modulation stems from a significant (negative) change in the real part of AlGaAs permittivity, which is maximum at band edge (Fig. 3c), boosted by the high quality-factor nonlocal resonance for TM-polarized light. The transient gain due to a negative change in the imaginary part of AlGaAs permittivity, achieved precisely in the same spectral range of the TM nonlocal resonance, further enhances the metasurface all-optical modulation performance. In this respect, our metasurface is active also in a more specific sense, i.e., it also behaves as an ultrathin optical amplifier capable of narrowing, on an ultrashort temporal window, the nonlocal resonance with which the probe signal is interacting.

A detailed comparison with relevant results previously reported on the concept of optical dichroism and birefringence modulation with metasurfaces using ultrafast lasers (see Supporting Information section [S3], Table S1) indicates, in a clear-cut way, the better performance of our approach in the visible and near infrared. In terms of power efficiency, we achieved a record high 6.7% transient dichroism (Δ*R/R*_TM_ – Δ*R/R*_TE_) per µJ cm^-2^ of pump fluence. This outperforms the results obtained by some of the present authors with a plasmonic metasurface (0.005% transient dichroism per µJ cm^-2^ pump fluence)^19^ mostly because of the much lower losses and thus higher quality factor enabled by semiconductor nanostructures. However, we reiterate the concept that such superior performance with semiconductor metasurfaces is enabled by a combination of factors, including, in particular, the precise tuning of a nonlocal resonance at the band-edge of AlGaAs, in order to benefit both from the very low material losses (enabling high quality factor resonances) and the intense permittivity modulation. This is ascertained by comparison with preliminary results achieved from a similar, though not optimized, AlGaAs-based metasurface design, where the dichroic performance was remarkable, but still 10 times lower^53^. The dramatic improvement achieved with the optimized configuration here demonstrated is also at the origin of the record high birefringence modulation, with a phase change and polarization rotation as large as 90°. Finally, it is worth noticing that, when considering other operation wavelengths, a similar dichroic/birefringence performance has been reported for metasurfaces operating in the 0.2–2.2 THz range^54^, but with much slower switching times (of about 1 ns) compared to our few ps modulation speed.

Although the operation mechanism behind our approach is intrinsically associated with a relatively (here ~15 nm) narrow bandwidth dictated by the spectral position of the band-edge, our design strategy can be easily extended to engineer AlGaAs-based devices operating efficiently on different spectral windows, since the bandgap energy can be tuned with Al percentage to be in the desired spectral range. Moreover, due to the generality of the described phenomena, our results suggest the versatility of direct-bandgap semiconductors as platforms for ultrafast light control. Engineering the interplay between band filling effect and resonances having even higher quality-factor can enhance the effects of all-optical modulations enabled by nonlinear metasurfaces. Lastly, an extension of our model to comprise not only transient amplification effects but also ultrafast light generation (spontaneous emission and amplified spontaneous emission) in AlGaAs nanomaterials can disclose for novel opportunities in the design of a broader class of active metadevices.

## Methods

### Experimental setup

The ultrafast transient dichroism and birefringence were measured using a home-built polarization-resolved pump-probe apparatus. The fundamental wavelength laser pulses, emitted at 800 nm with a temporal duration of 100 fs, were generated by a 1 kHz amplified Ti:sapphire laser system (Libra, Coherent). To produce the pump beam, we focused a portion of the fundamental laser output onto a β-barium borate (BBO) crystal, resulting in the generation of second harmonic pulses at 400 nm wavelength. The remaining fraction of the fundamental beam was directed into a near-IR optical parametric amplifier (OPA) to generate pulses centered at 1240 nm with an approximate bandwidth of 120 nm. The output of the OPA was then focused onto a YAG plate to create a broadband white light continuum probe beam, covering the spectral range of 550–950 nm. To control the relative time delay between the pump and probe pulses, we inserted an optical delay stage (PI Instruments) into the pump beam line and modulated the pump with a mechanical chopper (MC200B, Thorlabs) at a frequency of 500 Hz. Both the pump and probe beams were then non-collinearly focused on the sample with the probe beam incident at quasi-normal angles (*α* ~ 9°). The spot sizes on the sample were measured as Gaussian beam profiles with full width at half maximum (FWHM) values of 240 µm and 100 µm, respectively. After being reflected from the sample, the probe beam was re-collimated and directed into a visible-NIR spectrometer (Princeton Instruments) coupled with a linear photodetector array to record the differential spectral reflectance (Δ*R*/*R*) of the probe beam.

The polarization of the pump beam was fixed along the TM direction or 0° (defined with respect to the **k**_in_ direction, Fig. 1c inset) for all measurements. The pump beam angle of incidence, estimated to be around 15°, has a very minor quantitative impact on the all-optical modulation performance, due to the absence of high-quality resonances in this wavelength range. The pump fluence was set to 70 μJ cm^-2^ and 180 μJ cm^-2^ for the ultrafast transient dichroism and birefringence experiments, respectively.

For the birefringence experiment, note that the scheme presented in Fig. 1c is just a compact version of the full apparatus: a second mirror is placed after the sample, before the detection line, to properly direct the beam to the spectrometer. Thus, the measured polarization is resulting from the addition of the sample and the mirror effects.

### Modelling

The first step in our modelling approach describes the dynamics of the system internal degrees of freedom *n*_*1*_(*t*), *n*_*2*_(*t*) and *Θ*_L_(*t*). The 3TM is a slight modification of the one presented in refs. ^47,48^, considering that our system is a metasurface, that we modelled in 2D (with translational invariance in the direction of the wires length). We adopt the same reduced model approach to the carrier diffusion in the bulk of the structure, assuming that the diffusion is ambipolar and 1D from the top to the bottom of the structure. Since the nanowires have the same height as the pillars in ref. ^48^ and are excited at the same pump wavelength (400 nm), we set the same value of the characteristic diffusion time ${\tau }_{{\rm{d}}}=1.3$ ps^48^. We consider the diffusion and recombination processes to be segregated, with an effective recombination time ${\tau }_{{\rm{r}}}=8$ ps^48^.

The equations of the 3TM are as follows:

$$\frac{d{n}_{1}}{{dt}}=\frac{{qF}}{f\,{h}_{{skin}}}\frac{1}{h{\nu }_{P}}g(t)-\frac{{n}_{1}}{{\tau }_{d}}$$

$$\frac{d{n}_{2}}{{dt}}=\frac{{n}_{1}}{{\tau }_{d}}\frac{{h}_{{skin}}}{H}-\frac{{n}_{2}}{{\tau }_{r}}$$

$$\frac{d{\boldsymbol{\Theta }}_{L}}{{dt}}=\frac{{E}_{G}}{{c}_{L}}\frac{{n}_{2}}{{\tau }_{r}}$$

In the first equation, the drive term includes *q* = 0.58, the fraction of pump energy absorbed by the wire (calculated via full-wave simulations), *F*, the pump fluence, some geometric factors (*f* and *h*_skin_, the filling fraction of the active medium in the cell and the skin depth, respectively), the energy $h{\nu }_{P}$ of the pump photons, and the temporal profile *g*(*t*) of the pump pulse:

$$g(t)=\sqrt{\frac{4{ln}2}{\pi }}\frac{1}{{\tau }_{{FWHM}}}{exp} \left(-\frac{4{ln}2\,{t}^{2}}{{\tau }_{{FWHM}}^{2}}\right)$$

Here, ${\tau }_{{FWHM}}$ is the pulse full width at half maximum: in our case ${\tau }_{{\rm{FWHM}}}=100$ fs. The fluence in the simulations is reduced with respect to experiments (*F* = 35 μJ cm^-2^ and *F* = 100 μJ cm^-2^ for the dichroism and birefringence ones), to compensate for model overestimation^18^. Other relevant parameters are the bandgap energy ${E}_{G}=1.6528$ eV, estimated according to ref. ^22^ for AlGaAs with 18.5% Aluminum (nominal value 18%), the AlGaAs lattice heat capacity ${c}_{L}$ = 1.86 × 10^6 ^ J m^-3^ K^-1^ (ref. ^49^), and the height of the AlGaAs wires *H* = 400 nm.

The second step in our model consists in employing semiclassical formulas for the optical transitions in semiconductors. For the band filling effect, we follow the steps outlined in refs. ^46–48^. We work in the parabolic band approximation with contributions both from light holes (lh) and heavy holes (hh). Specifically, the population densities *n*_*1*_*(t)* and *n*_*2*_*(t)* contribute to modulating the absorption coefficient for probe wavelengths shorter than ${\lambda }_{G}=$750 nm according to

$$\Delta {\alpha }_{{n}_{i}}=\Delta {\alpha }_{{lh},{n}_{i}}+\Delta {\alpha }_{{hh},{n}_{i}}=\frac{{\lambda }_{{probe}}}{\sqrt{c}}\sqrt{\frac{1}{{\lambda }_{{probe}}}-\frac{1}{{\lambda }_{G}}}{({C}_{{lh}}G}_{{lh},{n}_{i}}({\lambda }_{{probe}})+{{C}_{{hh}}G}_{{hh},{n}_{i}}({\lambda }_{{probe}}))$$

where *c* is the speed of light in vacuum, ${C}_{{lh}}=3.85\times {10}^{13}$ m^-1^ s^-1/2^, ${C}_{{hh}}=7.81\times {10}^{13}$ m^-1^ s^-1/2^ are constants, dependent on AlGaAs material parameters^17^. Instead,

$${G}_{{lh},{n}_{i}}=F({E}_{{al}},{E}_{{F}_{V},{n}_{i}},T)-F({E}_{{bl}},{E}_{{F}_{c},{n}_{i}},T)-1$$

$${G}_{{hh},{n}_{i}}=F({E}_{{ah}},{E}_{{F}_{V},{n}_{i}},T)-F({E}_{{bh}},{E}_{{F}_{c},{n}_{i}},T)-1$$

where *F* is the Fermi−Dirac distribution at temperature *T*, corresponding to the lattice temperature; the quasi-Fermi levels ${E}_{{F}_{V},{n}_{i}}$, ${E}_{{F}_{C},{n}_{i}}$, which are carrier-dependent, and the energies ${E}_{{al}}$, ${E}_{{bl}}$, ${E}_{{ah}}$, ${E}_{{bh}}$ are evaluated according to equations 6a-6b, 8a-8b in ref. ^46^. The effective masses are *m*_*e*_ = 0.084m_0_, *m*_*lh*_ = 0.099m_0_, and *m*_*hh*_ = 0.573m_0_, for the electrons, light and heavy holes respectively, m_0_ being the free electron mass.

The modification of the absorption coefficient translates into a modulation of the imaginary part of the AlGaAs refractive index (N+iK) of $\Delta {K}_{{n}_{i}}={\lambda }_{{probe}}\Delta {\alpha }_{{n}_{i}}/2$. Kramers-Kronig formulas allow to retrieve the relative real part modulation $\Delta {N}_{{n}_{i}}$. Then the band filling permittivity modulation is

$$\Delta {\varepsilon }_{{{\rm{n}}}_{i}}^{{BF}}=2[N\Delta {N}_{{n}_{i}}-K\Delta {K}_{{n}_{i}}]+{\rm{i}}2[N\Delta {K}_{{n}_{i}}+K\Delta {N}_{{n}_{i}}]$$

The population densities *n*_*1*_*(t)* and *n*_*2*_*(t)* also contribute to permittivity modulation via a Drude-like mechanism,

$$\Delta {\varepsilon }_{{{\rm{n}}}_{i}}^{D}({\lambda }_{{probe}},t)={{Re}}(\Delta {\varepsilon }_{{{\rm{n}}}_{\rm{i}}}^{D})+{\rm{i}}{Im}(\Delta {\varepsilon }_{{{\rm{n}}}_{i}}^{D}):$$

$${{Re}}(\Delta {\varepsilon }_{{{\rm{n}}}_{i}}^{D})=-\frac{{e}^{2}{n}_{i}(t)}{{m}^{* }{\varepsilon }_{0}[{(2\pi c/{\lambda }_{{probe}})}^{2}+{\Gamma }_{{Drude}}^{2}]}$$

$${Im}(\Delta {\varepsilon }_{{{\rm{n}}}_{{\rm{i}}}}^{D})=-{Re}(\Delta {\varepsilon }_{{{\rm{n}}}_{i}}^{D})\frac{{\lambda }_{{probe}}{\Gamma }_{{Drude}}}{2\pi c}$$

Here, *e* is the electron charge, ${\varepsilon }_{0}$ the vacuum permittivity, *c* the speed of light in vacuum, and ${m}^{* }={(\frac{1}{{m}_{e}}+\frac{1}{{m* }_{h}})}^{-1}$ is the reduced mass for the electron-hole plasma, with ${m}_{h}^{* }$ defined as follows:

$${m}_{h}^{* }=\frac{{m}_{{hh}}^{3/2}+{m}_{{lh}}^{3/2}}{{m}_{{hh}}^{1/2}+{m}_{{lh}}^{1/2}}$$

The Drude damping term ${\Gamma }_{{\rm{Drude}}}$ has a value of $4.11\times {10}^{13}$ rad/s (which is of the order of the value reported in ref. ^48^ and estimated from GaAs carrier mobility), fitted on the experimental data.

Finally, the permittivity change caused by the lattice temperature increase with respect to the environment $\Delta {\Theta }_{{\rm{L}}}={\Theta }_{{\rm{L}}}-{T}_{{\rm{env}}}$, due to thermo-optic effects, can be expressed as ${\Delta \varepsilon }^{{TO}}({\lambda }_{{{probe}}},t)={Re}({\Delta \varepsilon }^{{TO}})+{\rm{i}}{Im}({\Delta \varepsilon }^{{TO}})$, where

$${Re}({\Delta \varepsilon }^{{TO}}({\lambda }_{{\rm{probe}}},t))=2[N({\lambda }_{{\rm{probe}}})\eta ]\Delta {\Theta }_{L}(t)$$

$${Im}({\Delta \varepsilon }^{{TO}}({\lambda }_{{\rm{probe}}},t))=2[K({\lambda }_{{\rm{probe}}})\eta ]\Delta {\Theta }_{L}(t)$$

The parameter $\eta$ is the AlGaAs thermo-optic coefficient, which is in general dispersed in wavelength, increasing as the band edge is approached. It is set to a constant mean value $\eta =1\times 1{0}^{-3}$ K^-1^ following the same approach as in ref. ^55^. Likewise, thermo-optic modulations of the imaginary refractive index were neglected.

The total, complex-valued Δε can be computed as the sum of these terms. For the free carriers, each of the populations *n*_*1*_*(t)* and *n*_*2*_*(t)* is responsible for permittivity modulations in the corresponding region of the structure (hot spots at the wire top and bulk, respectively). To simplify the calculations of the optical response, we divided the simulated wire geometry in two regions, a thin 16 nm layer on top, and the wire bulk below. In our calculations, the bulk experiences a modification due only to the second population, namely Δε = Δε(*n*_*2*_). On the contrary, we consider the top part of the wire to be subjected to both contributions, Δε = Δε(*n*_*1*_) + Δε(*n*_*2*_), as the *n*_*1*_ population gradually depletes. Plugging the values of the perturbed permittivity in our full-wave numerical simulation, we can retrieve the optical response as a function of time and probe wavelength. To this aim, we used commercial software COMSOL Multiphysics 6.1, employing finite element numerical methods to solve Maxwell equations; we set periodic Floquet boundary conditions on the sides of the unit cell, and port boundary conditions on the top-bottom edges (air and GaAs substrate). The simulated geometrical parameters, slightly differing from the nominal ones, are adjusted to match the static optical response: *W* = 150 nm, *H* = 400 nm, *P* = 400 nm. These discrepancies are likely due to fabrication defects on one hand, and to simulation limits on the other. Specifically, finite-size effects are expected to be particularly relevant, since the probe spot size was bigger than the patterned area of the sample. In this respect, we also take the substrate response into account, as detailed in the Supporting Information document, section S4.2. Lastly, we also included a rounding of the upper part of the wire and added a flat loss contribution of 0.03 to AlGaAs imaginary part of the permittivity, to mimic sample imperfections causing the broadening of the optical features.

### Polarization reconstruction

We report here the technique used for the unperturbed conditions; we proceeded similarly also for the dynamic phase reconstruction. See the Supporting Information document, section S5, for details on the error estimation process and statistical analysis.

To reconstruct the polarization of the reflected beam, we used a rotating quarter-waveplate and a polarizer, with the fast axis fixed at *γ* = 135° with respect to the ${\hat{{\boldsymbol{u}}}}_{{\bf{TM}}}$ direction in the polarization plane. We performed several measurements of the reflected beam intensity, corresponding to different angles of rotation β of the waveplate fast axis. Here, β is also defined with respect to ${\hat{{\boldsymbol{u}}}}_{{\bf{TM}}}$ and its values are *β* = [30°, 40°, 50°, 60°, 70°, 80°, 90°, 100°, 110°, 120°, 130°, 140°, 150°, 160°, 170°]. We employed groups of four of these measurements to solve the equation system presented in ref. ^56^ to obtain different estimates of the Stokes parameters I, M, C, S for each probe wavelength:

$$\begin{array}{l}R({\rm{\beta }},{\rm{\gamma }})=\frac{1}{2}\left\{{\rm{I}}+[{\rm{M}}\cos (2{\rm{\beta }})+{\rm{C}}\sin (2{\rm{\beta }})]\cos (2({\rm{\gamma }}-{\rm{\beta }}))\right.\\\left.\qquad\qquad+\,{\rm{S}}[\sin (2({\rm{\gamma }}-{\rm{\beta }}))]\right\}\end{array}$$

We then computed the phase $\varphi$ as a function of the C, S parameters. Lastly, we used statistical analysis to obtain the most reliable estimate for I, M, C, S and $\varphi$, from the ones obtained as mentioned and plotted the experimental polarization ellipse.

### Supplementary information

Supplemental Material

## Data Availability

All the data supporting this study are available upon request to the corresponding author.
