# Supplementary material for: Giant ultrafast dichroism and birefringence with active nonlocal metasurfaces
Source: Light Sci Appl. 2024 Aug 23;13:204. doi: 10.1038/s41377-024-01545-8 (PMC11344022; doi:10.1038/s41377-024-01545-8)
Supplement: Supplementary file 1 — Supplemental Material [file 41377_2024_1545_MOESM1_ESM.pdf]

Supporting Information for: Giant Ultrafast Dichroism and Birefringence with Active Nonlocal Metasurfaces

Giulia Crotti°[1,2], Mert Akturk°[1], Andrea Schirato[1], Vincent Vinel[3], Anton A. Trifonov[4], Ivan C. Buchvarov[4,5], Dragomir N. Neshev[6], Remo Proietti Zaccaria[2,7], Paolo Laporta[1,8], Aristide Lemaître[9], Giuseppe Leo[3,10], Giulio Cerullo[1,8], Margherita Maiuri[1,8], and Giuseppe Della Valle*[1,8]

[1]Department of Physics, Politecnico di Milano, 20133 Milano, Italy
[2]Istituto Italiano di Tecnologia, 16163, Genova, Italy
[3]Laboratoire Matériaux et Phénomènes Quantiques (MPQ), Université Paris Cité & CNRS, 75013, Paris, France
[4]John Atanasoff Center for Bio and Nano Photonics (JAC BNP), 1164 Sofia, Bulgaria
[5]Department of Physics, St. Kliment Ohridski University of Sofia, 5 James Bourchier Boulevard, 1164 Sofia, Bulgaria
[6]ARC Centre of Excellence for Transformative Meta-Optical Systems (TMOS), Research School of Physics, Australian National
[7]Cixi Institute of Biomedical Engineering, Ningbo Institute of Industrial Technology, Chinese Academy of Sciences, Ningbo 315201, China
[8]Istituto di Fotonica e Nanotecnologie (IFN), Consiglio Nazionale delle Ricerche, 20133, Milano, Italy
[9]Université Paris-Saclay, CNRS, Centre de Nanosciences et de Nanotechnologies, 10 Boulevard Thomas Gobert, 91120 Palaiseau, France
[10]Institut Universitaire de France (IUF)

S1 Fabrication

The sample was fabricated starting from the molecular-beam epitaxial growth of a 400 nm thick crystalline thin film of $Al_{.18}Ga_{.82}As$ on a 900 nm thick buffer of $Al_{.98}Ga_{.02}As$ on a [100] GaAs wafer. The $Al_{.18}Ga_{.82}As$ film was subsequently patterned in the form of the grating sketched in Fig. (1a) of the main document, first via electron-beam lithography (20keV Raith Pioneer Two hybrid SEM-EBL system) by making use of MaN2401 resist, and then via inductively coupled-plasma reactive-ion etching (Sentech SI500). Immediately after the etching, which reveals the aluminum-rich buffer, the latter undergoes

selective oxidation within a wet atmosphere at controlled temperature and pressure in a oven equipped with in-situ control (AET Technologies).

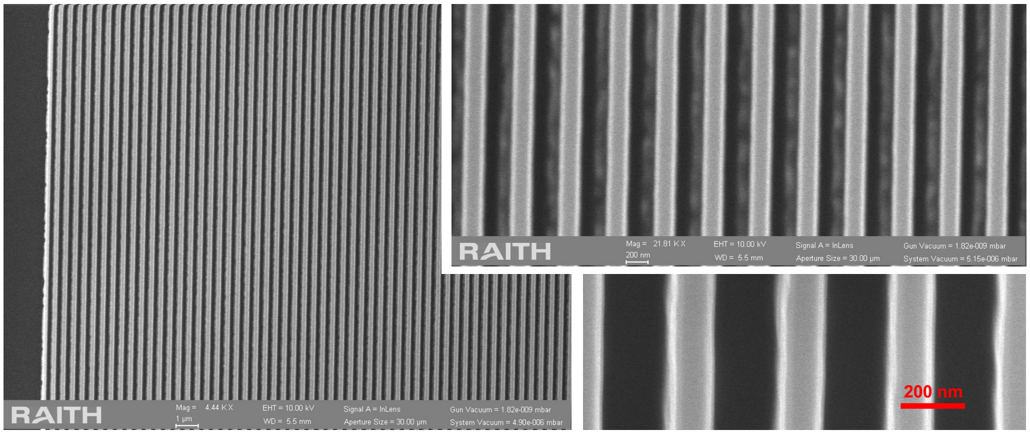

Figure S1: SEM images of an active nonlocal metasurface made of AlGaAs nanowires on AlO_x-GaAs substrate, representative of the sample used in our experiments. Nominal values of geometrical parameters: $W = 175$ nm, $P = 430$ nm, $H = 400$ nm.

While $Al_{.98}Ga_{.02}As$ oxidation results in the low-refractive-index ($n = 1.6$) that enables the necessary Mie-like resonances, such buffer is sandwiched between two 50 nm thick AlGaAs compositional tapers which grant mechanically stability to the whole structure after oxidation, despite the $\approx 11\%$ oxidation-induced linear shrinkage of the formerly lattice-matched $Al_{.98}Ga_{.02}As$ buffer. Greater details on the whole fabrication process can be found in Refs. [4, 9].

Scanning Electron Microscope (SEM) images of a sample representative of the quality of fabrication of the active nonlocal metasurface used in our experiments are shown in Fig. S1, at increasing resolutions (from left to right panels). Note the relatively good uniformity of the array on a spatial scale of tens of microns (left panel), showing the absence of macroscopic defects, even though a corrugation along the nanowire, corresponding to few nanometer roughness, is observed (right panel).

S2 Origin of the static reflection resonances

The origin of the resonances observed in the static, both TM and TE, reflectance spectra of the sample (refer to Fig. 1b in the main text) was ascertained by analysing numerically their dispersion. We performed scattering simulations for the unperturbed system in COMSOL, for a range of probe wavelengths and angles of incidence. Note that for this analysis, contrarily to the simulations discussed in the main text, we did not add any flat loss contribution. Besides, we completely neglected the contributions to reflection coming from substrate only (see section S4.2 of this document).

Figure S2 shows the calculated spectral and angular dispersions for TM (Fig. S1a) and TE (Fig. S1b) polarized light in reflection. Note the different ranges for the probe wavelength, centred on the TM and TE resonance, respectively.

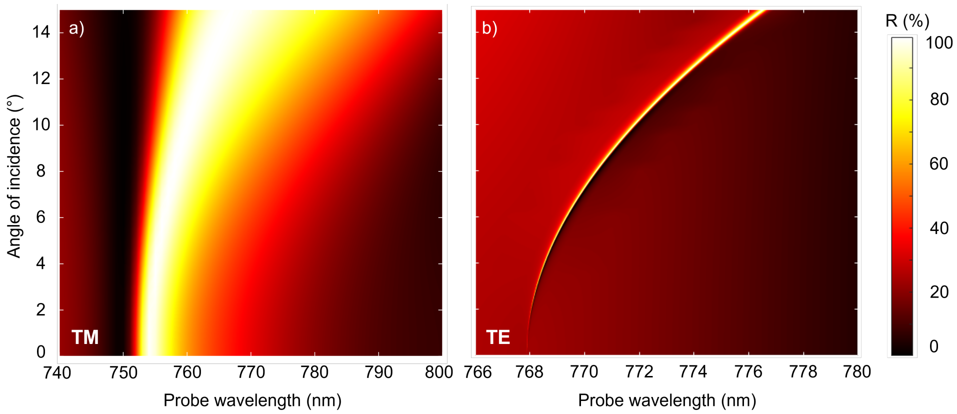

Figure S2: Simulated unperturbed reflectance to TM (a) and TE (b) polarized light, as a function of probe wavelength and angle of incidence. In (a), simulations were performed at steps of 0.5° and 1 nm, then linearly interpolated. In (b), at a selected angle of incidence, spectral resolution was increased around the resonance; results were afterwards interpolated using a parabolic fit for the dispersion.

The angular dispersion is pronounced for both polarizations, with a sizable shift of the peak with increasing angle of incidence. This indicates that the response of the metasurface is nonlocal. Indeed, the observed reflection resonances do not stem from a mode belonging to the single scatterer, but rather from a collective response of the periodical arrangement. For the TM resonance (Fig. S1a), along with the spectral shift, a broadening is also apparent. We identify this mode as a guided mode resonance, in light of its extended character and dispersive behaviour upon changes of the angle of incidence (i.e., the incoming light in-plane momentum). Given the possibility of exciting it even at a normal incidence, we do not interpret it as a symmetry-protected bound state in the continuum. This argument is also confirmed by further numerical simulations (not shown here), indicating that the metasurface under consideration does not exhibit infinite-Q factor TM eigenmodes at 758 nm, i.e. where the observed TM resonance sits.

In the TE case, the resonance quality factor diverges as we approach normal incidence, leading to a complete disappearance of the resonance at 0°. This is the signature of a symmetry-protected bound state in the continuum (BIC) [5]. The broadening of the resonance at higher angles is consistent with an increasing symmetry breaking dictated by the tilted illumination.

S3 Benchmarking the system performance

The performance of the present metasurface can be evaluated quantitatively from a comparison with previously reported results from literature. Table S1 provides an overview of related studies, collecting information on the employed platform, pump fluence, and corresponding modulations obtained.

The term $|\Delta_{\mathrm{TM}} - \Delta_{\mathrm{TE}}|$, with $\Delta_{\mathrm{TM,TE}} = \Delta R/R_{\mathrm{TM,TE}}$ or $\Delta_{\mathrm{TM,TE}} = \Delta T/T_{\mathrm{TM,TE}}$, measures the maximum transient dichroism, while the column $\Delta T(R)/T(R)$ indicates

Platform	Fluence ($\mu J\,cm^{-2}$)	$\lvert\Delta_{TM} - \Delta_{TE}\rvert$	$\frac{\Delta T(R)}{T(R)}$	$\Delta\varphi$	$\Delta\theta$	Ref.
GaAs metasurface	380	N/A	90%	N/A	N/A	[11]
a-Si:H metasurface	100	5%	5%	N/A	N/A	[3]
Au metamaterial	*800	*75%	*70%	30°	60°	[8]
CdO:In thin film	339	86%	*86%	N/A	50°	[14]
Au plasmonic crystal	N/A	N/A	85%	20°	N/A	[12]
Au metasurface	400	2%		N/A	N/A	[10]
ENZ material	10400	923%	960%	–	–	[13]
with Au metasurface	5100	–	–	26°	33°	
AlGaAs metasurface	70	40%	40%	N/A	N/A	[2]
AlGaAs metasurface	70	470%	470%	–	–	this work
	180	–	–	90°	90°	

Table S1: Comparison of the AlGaAs metasurface performance with relevant results from literature. Numbers with * are estimated from the figures reported in the papers, not stated explicitly by the authors. $\Delta_{TM,\,TE} = \Delta T(R)/T(R)$ for TM or TE linearly polarized light.

the maximum transmission or reflection modulations achieved in absolute value. Instead, $\Delta\varphi$ and $\Delta\theta$ are related to the birefringence modulation performance: $\Delta\varphi$ refers to the highest variation of the relative phase between the orthogonal components (see also fig. 4 in the main document), whereas $\Delta\theta$ measures the transient rotation of the polarization ellipse.

S4 Numerical simulations

S4.1 Model implementation

Figure S3 shows a schematic illustration of the algorithm used to retrieve numerically the optical observables to compare with the measurements outcome, i.e., $\Delta R/R$ and the reflected polarization ellipse, in the dichroism and birefringence experiments, respectively. The model inputs are the pump parameters, specifically its wavelength, temporal duration

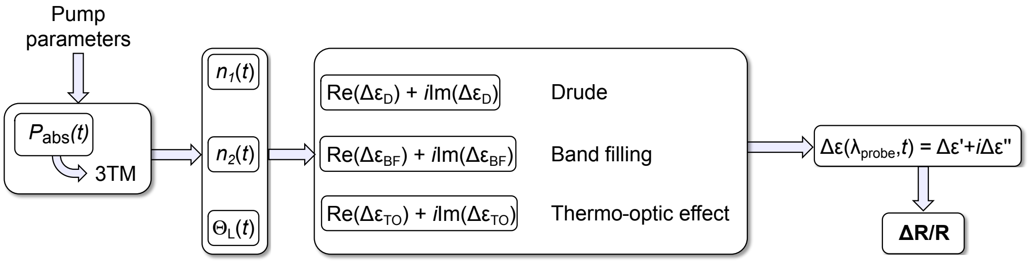

Figure S3: Flow chart of the modelling steps

and fluence. We started by estimating the absorbed pump power via a full-wave electromagnetic simulation of the metasurface: we solved Maxwell's equations in the frequency domain, by employing the Wave Optics module in COMSOL Multiphysics. The simulation geometry, represented in Fig. 1a of the main text, is the unit cell of the metasurface, with the following parameters: periodicity $P = 400\,\mathrm{nm}$, wire height $H = 400\,\mathrm{nm}$ and width $W = 150\,\mathrm{nm}$. As mentioned in the Methods section in the main text, these values are slightly modified with respect to the target fabrication parameters, to best fit the unperturbed optical response. Similarly, the simulated thickness of the AlO_x buffer is set to 890 nm. The upper part of the nanowire is rounded, with a fillet of 70 nm, to represent realistically the fabricated structure. The GaAs substrate and the air environment are 1350 nm and 1750 nm thick, respectively. At the lateral boundaries, Floquet conditions are enforced, while the plane-wave excitation is implemented via periodic ports. Specifically, the active port bounds the air domain on top, while the listener one is on the bottom, delimiting the substrate. The pump polarization is in-plane, i.e., the magnetic field $\mathbf{H}$ is along the z-direction. Moreover, the pump angle of incidence is set to 15°, according to the experimental conditions in the ultrafast pump-probe experiments. In this configuration, the fraction of the pump energy absorbed by the wire is ~ 0.58.

With the estimated absorption, we had all the parameters needed for the second step of the modelling algorithm, namely the integration of the 3TM for the dynamical degrees of freedom $n_1(t)$, $n_2(t)$ and $\Theta_L(t)$ (see Methods section of the main text). To this aim, we employed the `ode45` solver in MATLAB [6], which is based on a Runge-Kutta formula.

Note that in general, according to the pump excitation conditions, specific spatial patterns of the hot spots are induced across the wire. As such, our assumption of a 1D diffusion of hot carriers from the top region down to the bulk could lead to e.g. overestimating the peak delay of the bulk carrier population $n_2(t)$ and corresponding optical modulation. The use of an effective diffusion constant D to estimate the diffusion timescale τ_D (in agreement with ref. [7]) is expected to mitigate the effects of resorting to such a reduced model approach. Possible extensions of the model, to account for the full 2D diffusion process, could be envisaged, starting from the modification of the 3TM equations with the addition of spatial dependencies and explicit diffusion terms. A carrier-dependent mobility or recombination could also be considered as further refinements of the model. This formulation would, however, considerably increase the computational effort and is out of the scope of this work. Indeed, the reduced approach is revealed to be accurate in the reconstruction of the temporal evolution of the transient optical signal, without the need of employing fitting parameters.

The following step is the computation of the permittivity variation as a function of the dynamical degrees of freedom and of the relevant probe wavelengths between 700 nm and 800 nm, that we performed by implementing the analytic formulas in MATLAB. More specifically, we defined two variables, $\Delta\varepsilon_{\mathrm{bulk}}$ and $\Delta\varepsilon_{\mathrm{skin}}$ for the bulk and hot-spot regions, respectively. As specified in the main text, the former is computed by considering the hot carrier phenomena caused by the presence of the $n_2(t)$ population only, plus the lattice effects: $\Delta\varepsilon_{\mathrm{bulk}} = \Delta\varepsilon^{D}_{n_2} + \Delta\varepsilon^{BF}_{n_2} + \Delta\varepsilon^{TO}$, where the superscripts D, BF and TO refer to the Drude, band filling and thermo-optic contributions. In the calculations for determining the hot-spot region permittivity modulation, instead, both populations are included. Thus, $\Delta\varepsilon_{\mathrm{skin}} = \Delta\varepsilon^{D,BF}_{n_1} + \Delta\varepsilon^{D,BF}_{n_2} + \Delta\varepsilon^{TO}$.

Having computed the total permittivity changes, we then performed full-wave calculations to simulate the optical response of the out-of-equilibrium structure, at each

probe wavelength and time delay. We used the LiveLink interface between COMSOL and MATLAB to set the perturbed permittivity and run the simulations. The geometry of the unit cell is identical to the one described above, except for an additional division of the wire geometry, in two regions: the bulk and the hot-spot domains, obtained by defining a segment, isolating a 16 nm-thick strip on the top part of the structure. In these domains, the permittivity modulation was set to be $\Delta\varepsilon_{\text{bulk}}$ and $\Delta\varepsilon_{\text{skin}}$ respectively. The simulation geometry is shown in Figure S4. As for the electromagnetic simulations in static conditions, Maxwell's equations were solved in the frequency domain in the Wave Optics module; the boundary conditions were Floquet periodic along the vertical edges, with ports along the top and bottom boundaries. Pure TE and TM polarizations were set for the dichroism simulations, whereas mixed polarization (linear, at 135° in the TM-TE plane) was imposed for the birefringence case. From these computations, the total reflection and, if relevant, the Jones vector representing the reflected polarization were extracted. These data were used to derive the quantities to be compared with the measurements, taking also into account the role of the substrate as explained in the section below.

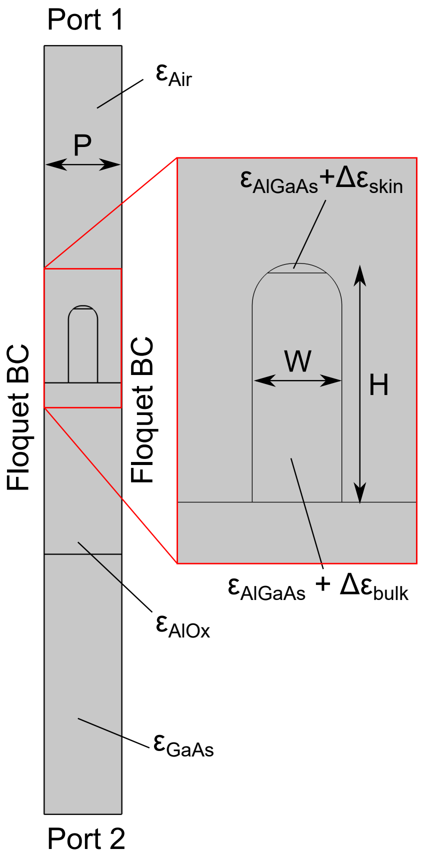

Figure S4: Geometry of the unit cell for the simulations of the out-of-equilibrium structure. On the right, the zoomed view of the nanowire.

S4.2 The role of substrate

As mentioned in the main text, the probe spot-size at FWHM is $\sim 100\,\mu$m. The nanowires array is instead a $70\,\mu\text{m} \times 70\,\mu\text{m}$ square: therefore, the unpatterned substrate contributes to the optical response, for a fraction of the reflected light, both in unperturbed and perturbed conditions. In the simulations, we took this detail into account as follows.

First, we assumed that the optical pumping has practically no effect on the substrate, so that the $\Delta R/R$ signal comes almost entirely from the perturbation of the metasurface optical response. This hypothesis was corroborated by additional pump-probe measurements (not reported here) performed on the AlOx-GaAs substrate only, which revealed a modulation of reflection of less than 2%. Therefore, we neglected this modification in the simulations, and considered the substrate reflectivity as unaltered at all time delays following pump arrival.

Thus, the dynamic reflectivity of the whole illuminated system, i.e. including the nanowires array (referred to as the sample) plus substrate (unpatterned GaAs-AlOx only), at probe wavelength λ and time delay t can be written as

$$R(\lambda, t) = rR_{\text{sample}}(\lambda, t) + (1 - r)R_{\text{substrate}}(\lambda), \tag{S1}$$

where $r \in [0, 1]$ is a number representing the ratio between the metasurface area and the illuminated one. For the two experiments, this implies a correction on the simulated quantities, to properly compare with experimental measurements.

Dichroism experiment The figure of merit in this case is $\Delta R/R$. By direct substitution of eq. (S1),

$$\frac{\Delta R}{R} = \frac{R(\lambda, t) - R(\lambda, 0)}{R(\lambda, 0)} = \frac{rR_{\text{sample}}(\lambda, t)}{rR_{\text{sample}}(\lambda, t) + (1 - r)R_{\text{substrate}}(\lambda)}.$$

To compute numerically this quantity, beside retrieving $R_{\text{sample}}(\lambda, t)$, it is sufficient to compute $R_{\text{substrate}}(\lambda)$ via a full-wave electromagnetic simulation. The parameter r is fitted on the experimental data, $r = 0.52$, which is close to a geometrical estimate $(70\,\mu\text{m})^2/\pi(50\,\mu\text{m})^2 = 0.62$.

Birefringence experiment Reconstruction of polarization from simulated quantities is slightly complicated by the correction introduced in eq. (S1).

We first computed, via full-wave simulations in COMSOL, the Jones vector representing the polarization state of the wave reflected by the sample, along with the reflectivity $R_{\text{sample}}(\lambda, t)$. We then took into account the detection line and computed the expected output reflectivity $R_{\text{sample}}(\lambda, t; \gamma, \beta)$ via Jones matrix calculus. The same was done for the substrate; we then derived the total output reflectivity by summing sample and substrate contributions, weighted by r and $(1 - r)$, respectively. In this case, $r = 0.65$ (corresponding to a slightly smaller probe) and the angle of incidence used in the simulation was 7° (slightly modified with respect to the nominal experimental one of 9°). Finally, we solved equation (S2) and retrieved the Stokes parameters.

S4.3 Contributions to permittivity modulation

To make it possible to fully appreciate the relative weights of the effects presiding over the third-order nonlinearity in our system, we include here a more detailed version (Fig. S5)

of Figure 3c of the main document. In the panels from left to right, the contributions from Drude, band filling and thermo-optic effects to the bulk $\Delta\varepsilon$ are plotted, at a time delay of $t = 2\,\text{ps}$.

From the comparison of the scales on the respective y-axes, notice that the real permittivity perturbation induced by band filling is one and two orders of magnitude greater than the ones corresponding to Drude and thermo-optic phenomena, respectively; the contrast is even higher for the imaginary part of $\Delta\varepsilon$ in the 700–750 nm range of the spectrum, as mentioned in the main document. As it describes the modulation of interband optical transitions, due to the fact that the lower part of conduction band has been populated upon pump absorption, band filling is dominant and peaks at probe wavelengths near bandgap (750 nm). In comparison, the other effects are only mildly dispersed in the same spectral region. It is worth pointing out that the lattice temperature increase gives rise to a positive $\text{Re}(\Delta\varepsilon_{\text{TO}})$ (see the right panel), consistent with the positive value of the thermo-optic coefficient η.

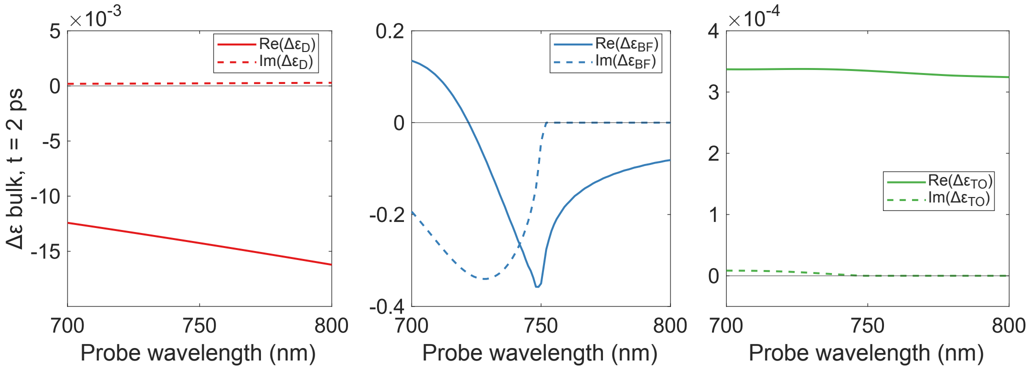

Figure S5: Permittivity variations in the nanowires bulk at $t = 2\,\text{ps}$. From left to right, the contributions coming from Drude, band filling and thermo-optic effects. Solid (dashed) lines correspond to the real (imaginary) part of $\Delta\varepsilon$.

S4.4 Lattice temperature

We also complemented our simulation of the ultrafast response of the system by modelling the dynamical variables evolution over longer timescales to properly resolve the complete relaxation of the hot carriers towards the lattice (reached within $\sim 40\,\text{ps}$ in our simulation); specifically, we solved the 3TM for time delays of up to 100 ps, and examined the lattice temperature increase as a function of time. In this way, we estimated the maximum $\Delta\Theta_L$ induced by the single pump pulse in the birefringence experiment, which featured the highest value of pump fluence. The results are reported in fig. S6. We notice that, due to the low (kHz) repetition rate of the laser, cumulative effects on the sample temperature can be neglected. It is important to highlight that the extracted $\Delta\Theta_{\text{L,MAX}} \sim 1.2\,\text{K}$ is a conservative estimate since the employed 3TM does not include a coupling term between the lattice and the external environment, thus neglecting cooling of the nanowires (see the temperature plateau at longer times). Thus, our model excludes the possibility that the temperature increase could be responsible for permanent damage. Most importantly, this

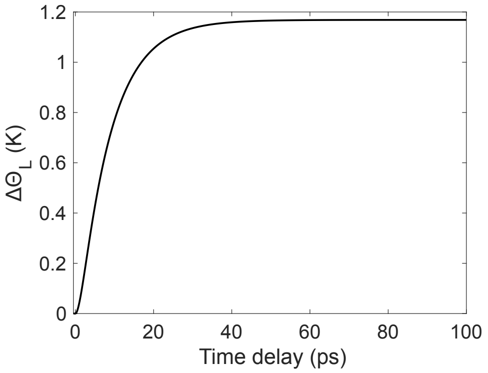

Figure S6: Predicted evolution of $\Delta\Theta_L$, the increase of lattice temperature with time, for the birefringence experiment (simulated fluence $\sim 100\,\mu\mathrm{J\,cm^{-2}}$).

is corroborated by the fact that the birefringence measurements were repeated several times, with similar excitation conditions, and no sample degradation was observed.

S5 Polarization reconstruction

S5.1 Static polarization

Stokes parameters

As mentioned in the main text, in order to estimate the polarization state of the beam reflected by the sample, we solved, at a selected probe wavelength, for the Stokes parameters (I, M, C, S) in the following system of equations [1]:

$$R(\beta,\gamma) \;=\; \frac{1}{2}I + [M\cos(2\beta) + C\sin(2\beta)]\cos(2(\gamma-\beta)) + S[\sin(2(\gamma-\beta))]. \qquad \text{(S2)}$$

Here, $R(\beta,\gamma)$ is the measured reflection in the configuration of angles γ and β respectively of the polarizer and quarter-waveplate fast axis in the polarization plane (see Fig. 1c in the main text). We fixed $\gamma = 135°$, and repeated the measurements for fifteen distinct values of β, ranging from 30° to 170° in steps of 10°, i.e.:

$$\beta = [30°, 40°, 50°, 60°, 70°, 80°, 90°, 100°, 110°, 120°, 130°, 140°, 150°, 160°, 170°] \qquad \text{(S3)}$$

A combination of four of these measurements allows to retrieve an estimate of the Stokes parameters. The solution amounts to invert the β-dependent matrix $\mathbf{H}$ implicitly defined on the right-hand side of the equation (S2), namely

$$\begin{pmatrix} I \\ M \\ C \\ S \end{pmatrix} = \mathbf{H}^{-1}_{[\beta_1,\beta_2,\beta_3,\beta_4]} \begin{pmatrix} R(\gamma,\beta_1) \\ R(\gamma,\beta_2) \\ R(\gamma,\beta_3) \\ R(\gamma,\beta_4) \end{pmatrix}. \qquad \text{(S4)}$$

The error on each of the Stokes parameters $(\sigma_I^2, \sigma_M^2, \sigma_C^2, \sigma_S^2)$ depends on the degree of accuracy of each reflection measurement, but also on the choice of the combination itself. This is clear upon propagation of the uncertainty of R onto I, M, C, S from eq. (S4); the dependence on the chosen β_{1-4} parameters through $\mathbf{H}^{-1}$ is manifest. We define the optimal combination of β angles as the one which minimises the sum $\sigma_I^2 + \sigma_M^2 + \sigma_C^2 + \sigma_S^2$, without considering the cross-terms (such as σ_{IM} or σ_{CS}) for simplicity. Intuitively, the optimal configurations correspond to the ones having well-separated values of β.

In order to both exploit all information from experiments, and maintain the statistical analysis agile, we grouped the measurements as follows. We selected the best configuration according to the aforementioned criterion, and obtained the set corresponding to $\beta_{\text{set1}} = [30°, 60°, 100°, 170°]$. Then, the second best was selected, excluding the measurements already appearing in the first choice: $\beta_{\text{set2}} = [50°, 80°, 110°, 160°]$. The same was done for the third, $\beta_{\text{set3}} = [70°, 90°, 130°, 150°]$. For the last configuration, we grouped the remaining three datasets and selected the fourth one according again to the criterion mentioned above, thus obtaining $\beta_{\text{set4}} = [40°, 120°, 140°, 170°]$. In this way, it was possible to extract four different estimates for (I, M, C, S) based on each of these sets. Notice that since the measurements corresponding to $\beta = 170°$ appears in both the first and fourth one, these estimates *are not independent*.

This implies that, when computing the weighted averages to obtain the best estimates $(I_{\text{best}}, M_{\text{best}}, C_{\text{best}}, S_{\text{best}})$, correlations have to be taken into account. Consider for example the four different values $(I_{\text{set1}}, I_{\text{set2}}, I_{\text{set3}}, I_{\text{set4}})$. Each of these is a linear function of the reflection measurements from the corresponding set, namely, for example:

$$I_{\text{set1}} = H^{-1}_{[\beta_{\text{set1}}]11} R(\gamma, \beta = 30°) + H^{-1}_{[\beta_{\text{set1}}]12} R(\gamma, \beta = 60°) + H^{-1}_{[\beta_{\text{set1}}]13} R(\gamma, \beta = 100°) + H^{-1}_{[\beta_{\text{set1}}]14} R(\gamma, \beta = 170°)$$

Let $\mathbf{A}$ be the 4-by-15 matrix with entries defined by

$$A_{jk} = \frac{\partial I_{\text{set}j}}{\partial[R(\gamma, \beta(k))]}, \tag{S5}$$

where the index j runs on the four sets of measurements, whereas the index k refers to the values of β, i.e. the array in eq. (S3). $\mathbf{A}$ is a sparse matrix, since in the j-th row the only nonzero elements are the ones corresponding to the four β values in the set j. Their values correspond to the entries in the first row of $\mathbf{H}^{-1}_{[\beta_{\text{set}j}]}$. Then, the 4-by-4 covariance matrix for the derived quantities $\mathbf{I} = (I_{\text{set1}}, I_{\text{set2}}, I_{\text{set3}}, I_{\text{set4}})$ is defined by

$$\mathbf{\Sigma^I} = \mathbf{A \Sigma^R A}^T, \tag{S6}$$

where $\mathbf{\Sigma^R}$ is the 15-by-15 covariance matrix of the reflection measurements. In our case, we consider the measurements to be uncorrelated and having the same variance of $(0.01)^2$, so that $\mathbf{\Sigma^R}$ is proportional to the identity, $\mathbf{\Sigma^R} = (0.01)^2\mathbb{I}$.

The resulting $\mathbf{\Sigma^I}$ has diagonal terms corresponding to the variance of each estimate of I; besides, it has two non-zero off-diagonal entries, (1,4) and (4,1), accounting for the correlation between I_{set1} and I_{set4}.

With $\mathbf{\Sigma^I}$ at hand, it is straightforward to compute the uncertainty $\sigma^2_{I_{\mathrm{best}}}$:

$$\sigma^2_{I_{\mathrm{best}}} = \left(\begin{pmatrix} 1 & 1 & 1 & 1 \end{pmatrix} \mathbf{\Sigma^I}^{-1} \begin{pmatrix} 1 \\ 1 \\ 1 \\ 1 \end{pmatrix} \right)^{-1}, \tag{S7}$$

and the weighted average I_{best} itself:

$$I_{\mathrm{best}} = \sigma^2_{I_{\mathrm{best}}} \left(\begin{pmatrix} 1 & 1 & 1 & 1 \end{pmatrix} \mathbf{\Sigma^I}^{-1} \mathbf{I}^T \right). \tag{S8}$$

Treatment of the other Stokes parameters is analogous, providing M_{best}, C_{best}, S_{best}. With this estimate of the Stokes parameters at hand, the polarization ellipse can be plotted. The Jones vector analysis, which is completely equivalent to the Stokes representation, can also be easily derived. However, since we were interested in using $\varphi = \varphi_{\mathrm{TM}} - \varphi_{\mathrm{TE}}$, the relative phase between the components, as a figure of merit, we derived it directly following the steps outlined below.

Relative phase φ

Employing the same sign convention as in [1] for the S parameter, it is simple to derive

$$\varphi = \mathrm{atan2}(-S, C) \equiv \arg[C + \mathrm{i}(-S)], \tag{S9}$$

which corresponds to $\arctan(-S/C)$ if $C > 0$. With this definition, φ takes values in the interval $(-\pi, \pi]$. Notice however that, since φ represents an angle, it can be equivalently written as a number in $[0, 2\pi)$. In particular, in figure 4 of the main text (panels a, b) we chose this last representation to make the images more readable.

Therefore, to derive an estimate of φ using all information from measurements, we decided to adopt the following method. Given

$$\mathbf{C} = (C_{\mathrm{set1}}, C_{\mathrm{set2}}, C_{\mathrm{set3}}, C_{\mathrm{set4}})$$

and

$$\mathbf{S} = (S_{\mathrm{set1}}, S_{\mathrm{set2}}, S_{\mathrm{set3}}, S_{\mathrm{set4}})$$

from the previous steps, since φ is a (nonlinear) function of C, S, we can estimate it by considering the couples in a subset of

$$\mathbf{C} \times \mathbf{S} = \{(C_{\mathrm{set1}}, S_{\mathrm{set1}}), (C_{\mathrm{set1}}, S_{\mathrm{set2}}), \ldots, (C_{\mathrm{set2}}, S_{\mathrm{set1}}), \ldots\}.$$

We chose one of the maximal subsets (exploiting all the fifteen measurements without any redundancy), specifically

$$\{(C_{\mathrm{set1}}, S_{\mathrm{set1}}), (C_{\mathrm{set1}}, S_{\mathrm{set2}}), (C_{\mathrm{set1}}, S_{\mathrm{set3}}), (C_{\mathrm{set1}}, S_{\mathrm{set4}}), (C_{\mathrm{set2}}, S_{\mathrm{set1}}), \\ (C_{\mathrm{set3}}, S_{\mathrm{set1}}), (C_{\mathrm{set4}}, S_{\mathrm{set1}})\},$$

and we got 7 different estimates: $\boldsymbol{\varphi} = (\varphi_1, \varphi_2, \cdots, \varphi_7)$. To average them, it is necessary to find the covariance matrix $\mathbf{\Sigma}^{\varphi}$, since they are correlated. Following the method of

moments, as a first step we need to compute the covariance matrix $\mathbf{\Sigma^{CS}}$ with entries

$$\Sigma_{jk}^{CS} = \begin{cases} \sigma_{C_{\text{set}l}C_{\text{set}m}}, \; l = \dfrac{j-1}{2}+1, m = \dfrac{k-1}{2}+1 & \text{if } j,k \text{ odd} \\ \sigma_{C_{\text{set}l}S_{\text{set}m}}, \; l = \dfrac{j-1}{2}+1, m = \dfrac{k}{2} & \text{if } j \text{ odd and } k \text{ even} \\ \sigma_{S_{\text{set}l}C_{\text{set}m}}, \; l = \dfrac{j}{2}, m = \dfrac{k-1}{2}+1 & \text{if } j \text{ even and } k \text{ odd} \\ \sigma_{S_{\text{set}l}S_{\text{set}m}}, \; l = \dfrac{j}{2}, m = \dfrac{k}{2} & \text{if } j,k \text{ even} \end{cases}$$

This is done by considering the matrix $\mathbf{A'}$, similar to $\mathbf{A}$ defined in eq. (S5), but containing both the derivatives of $\mathbf{C}$ and $\mathbf{S}$ with respect to $R(\gamma,\beta)$, so that

$$\mathbf{\Sigma^{CS}} = \mathbf{A'}\mathbf{\Sigma^{R}}\mathbf{A'}^{T}.$$

$\mathbf{\Sigma^{CS}}$ is an 8×8 matrix, which is almost block diagonal, since correlations are nonzero only for C, S of the same set and between the C, S of the first and fourth set. The following step consists in computing the Jacobian $\mathbf{J}$ of the functions $\boldsymbol{\varphi}$ with respect to $\mathbf{C},\mathbf{S}$ such that

$$J_{jk} = \begin{cases} \dfrac{\partial\varphi_j}{\partial C_{\text{set}l}}, \; l = \dfrac{(k-1)}{2}+1 & \text{if } k \text{ odd} \\ \dfrac{\partial\varphi_j}{\partial S_{\text{set}l}}, \; l = \dfrac{k}{2} & \text{if } k \text{ even} \end{cases}$$

Finally,

$$\mathbf{\Sigma}^{\varphi} = \mathbf{J}\mathbf{\Sigma^{CS}}\mathbf{J}^{T} \tag{S10}$$

and the error propagation on $\boldsymbol{\varphi}$ gives

$$\sigma^2_{\varphi_{\text{best}}} = \left(\begin{pmatrix} 1 & 1 & 1 & 1 \end{pmatrix} \mathbf{\Sigma}^{\varphi\,-1} \begin{pmatrix} 1 \\ 1 \\ 1 \\ 1 \end{pmatrix} \right)^{-1}. \tag{S11}$$

The weighted average is therefore

$$\varphi_{\text{best}} = \sigma^2_{\varphi_{\text{best}}} \left(\begin{pmatrix} 1 & 1 & 1 & 1 \end{pmatrix} \mathbf{\Sigma}^{\varphi\,-1} \boldsymbol{\varphi}^{T} \right). \tag{S12}$$

S5.2 Dynamic polarization

For the reconstruction of the dynamic polarization, the first step was to obtain the reflection $R'(\lambda,t)$ at each probe wavelength and time delay, starting from the pump-probe signal $\Delta R/R$. This can be done easily according to the formula $R'(\lambda,t) = [\Delta R/R(\lambda,t)]R(\lambda) + R(\lambda)$. The uncertainty on $R'(\lambda,t)$ is derived with error propagation, by considering an error of 1% on the pump-probe signal. Then, each step mentioned in the previous section was repeated at every probe wavelength and time delay.

References

[1] H. G. Berry, G. Gabrielse, and A. E. Livingston. "Measurement of the Stokes parameters of light". In: *Applied Optics* 16.12 (Dec. 1977), p. 3200. DOI: 10.1364/ao.16.003200. URL: https://doi.org/10.1364/ao.16.003200.

[2] Giulia Crotti et al. "Anisotropic Metasurface for Ultrafast Polarization Control via All-Optical Modulation". In: *Proceedings of the 11th International Conference on Photonics, Optics and Laser Technology*. SCITEPRESS - Science and Technology Publications, 2023. DOI: 10.5220/0011926600003408. URL: http://dx.doi.org/10.5220/0011926600003408.

[3] Giuseppe Della Valle et al. "Nonlinear Anisotropic Dielectric Metasurfaces for Ultrafast Nanophotonics". In: *ACS Photonics* 4.9 (Sept. 2017), pp. 2129–2136. ISSN: 2330-4022. DOI: 10.1021/acsphotonics.7b00544. URL: http://dx.doi.org/10.1021/acsphotonics.7b00544.

[4] V. F. Gili et al. "Monolithic AlGaAs second-harmonic nanoantennas". In: *Optics Express* 24.14 (July 2016), p. 15965. ISSN: 1094-4087. DOI: 10.1364/oe.24.015965. URL: http://dx.doi.org/10.1364/OE.24.015965.

[5] Chia Wei Hsu et al. "Bound states in the continuum". In: *Nature Reviews Materials* 1.9 (July 2016). ISSN: 2058-8437. DOI: 10.1038/natrevmats.2016.48. URL: http://dx.doi.org/10.1038/natrevmats.2016.48.

[6] The MathWorks Inc. *MATLAB version: 9.13.0 (R2022b)*. Natick, Massachusetts, United States, 2022. URL: https://www.mathworks.com.

[7] Andrea Mazzanti et al. "All-Optical Modulation with Dielectric Nanoantennas: Multiresonant Control and Ultrafast Spatial Inhomogeneities". In: *Small Science* 1.7 (May 2021). ISSN: 2688-4046. DOI: 10.1002/smsc.202000079. URL: http://dx.doi.org/10.1002/smsc.202000079.

[8] Luke H. Nicholls et al. "Ultrafast synthesis and switching of light polarization in nonlinear anisotropic metamaterials". In: *Nature Photonics* 11.10 (Sept. 2017), pp. 628–633. ISSN: 1749-4893. DOI: 10.1038/s41566-017-0002-6. URL: http://dx.doi.org/10.1038/s41566-017-0002-6.

[9] Mohsen Rahmani et al. "Nonlinear frequency conversion in optical nanoantennas and metasurfaces: materials evolution and fabrication". In: *Opto-Electron Adv* 1.10 (2018), pp. 180021-1-180021–12. ISSN: 2096-4579. DOI: 10.29026/oea.2018.180021.

[10] Andrea Schirato et al. "Transient optical symmetry breaking for ultrafast broadband dichroism in plasmonic metasurfaces". In: *Nature Photonics* 14.12 (Oct. 2020), pp. 723–727. ISSN: 1749-4893. DOI: 10.1038/s41566-020-00702-w. URL: http://dx.doi.org/10.1038/s41566-020-00702-w.

[11] Maxim R. Shcherbakov et al. "Ultrafast all-optical tuning of direct-gap semiconductor metasurfaces". In: *Nature Communications* 8.1 (May 2017). ISSN: 2041-1723. DOI: 10.1038/s41467-017-00019-3. URL: http://dx.doi.org/10.1038/s41467-017-00019-3.

[12] Mohammad Taghinejad et al. “Ultrafast Control of Phase and Polarization of Light Expedited by Hot-Electron Transfer”. In: *Nano Letters* 18.9 (Aug. 2018), pp. 5544–5551. ISSN: 1530-6992. DOI: 10.1021/acs.nanolett.8b01946. URL: http://dx.doi.org/10.1021/acs.nanolett.8b01946.

[13] Kuidong Wang et al. “High Contrast, Femtosecond Light Polarization Manipulation in Epsilon-near-Zero Material Coupled to a Plasmonic Nanoantenna Array”. In: *ACS Photonics* 8.9 (Sept. 2021), pp. 2791–2799. ISSN: 2330-4022. DOI: 10.1021/acsphotonics.1c00971. URL: http://dx.doi.org/10.1021/acsphotonics.1c00971.

[14] Yuanmu Yang et al. “Femtosecond optical polarization switching using a cadmium oxide-based perfect absorber”. In: *Nature Photonics* 11.6 (May 2017), pp. 390–395. ISSN: 1749-4893. DOI: 10.1038/nphoton.2017.64. URL: http://dx.doi.org/10.1038/nphoton.2017.64.
